# Optimizing Muscle Quality in Common Carp (*Cyprinus carpio* L.): Impacts of Body Size on Nutrient Composition, Texture, and Volatile Profile

**DOI:** 10.3390/foods14162794

**Published:** 2025-08-11

**Authors:** Zijie He, Junli Wang, Yun Wei, Xiao Yan, Yuanyou Li, Dizhi Xie, Guoxing Nie

**Affiliations:** 1 College of Fisheries, Henan Normal University, Xinxiang 453007, China; jie3719@126.com (Z.H.); 15238906698@163.com (Y.W.); yanxiaobangong@163.com (X.Y.); 2 College of Life Sciences, Henan Normal University, Xinxiang 453007, China; wangjunli1971@126.com; 3 College of Marine Sciences, South China Agricultural University, Guangzhou 510642, China; yyli16@scau.edu.cn

**Keywords:** *Cyprinus carpio*, cultivated, LC-PUFA deposition, volatile flavor compounds, nutritional value and texture, ontogenetic development

## Abstract

To investigate the effect of body size on muscle quality of common carp (*Cyprinus carpio* L.), we systematically tracked the dynamic changes in nutrient content, texture, and volatile organic compounds (VOCs) among small-sized (~100 g), medium-sized (~250 g), and large-sized (~600 g) fish (SYRC, MYRC, and HYRC, respectively) over a 30-week feeding trial. The results indicated that the HYRC showed significantly reduced moisture and lipid content, along with increased protein content, hydroxyproline, hardness, and chewiness compared to the SYRC (*p* < 0.05). The long-chain polyunsaturated fatty acids (LC-PUFAs) and fish lipid quality in the MYRC were significantly lower than those in both the SYRC and HYRC (*p* < 0.05). The HYRC demonstrated an elevated health-promoting index and a reduced atherogenicity value compared to the SYRC (*p* < 0.05). The contents of alcohol, ketones, and furans in the HYRC increased by 32.53%, 44.62%, and 144.29%, respectively, compared with those in the SYRC (*p* < 0.05), including key VOCs in aquatic products such as oct-1-en-3-ol and pent-1-en-3-ol. In conclusion, the SYRC have higher levels of LC-PUFAs and lower hardness; the MYRC have poor levels of LC-PUFAs; and the HYRC have an optimal synergy of nutrition, texture, and VOCs, but the overaccumulation of undesirable VOCs requires mitigation. This provides theoretical references and data support for fish quality optimization, processing, and consumption guidelines.

## 1. Introduction

Fish hold an indispensable position in the human diet due to their unique and highly optimized nutritional composition. Their core value lies not only in providing high-bioavailability protein but also in serving as a primary source of long-chain polyunsaturated fatty acids (LC-PUFAs), such as eicosapentaenoic acid (EPA, 20:5n-3) and docosahexaenoic acid (DHA, 22:6n-3), which are scarce in terrestrial foods [1,2]. These components are critically important for cardiovascular health, neurodevelopment, and metabolic regulation [3,4,5]. Consequently, from a nutritional functionality perspective, the nutritional support provided by fish transcends their role as mere sources of energy or protein. This establishes their unique and vital core position in balancing dietary patterns, preventing nutrition-related chronic diseases, and safeguarding the health of populations across all age groups, particularly infants, young children, and elderly populations [6].

Despite the remarkable nutritional value of fish, global aquaculture practices over the past decades have predominantly prioritized growth rate and disease resistance as core evaluation metrics, operating under a long-standing “yield-first” paradigm [7]. Alongside societal development and rising consumer demand, market demand for high-quality aquatic products has surged, driving a strategic shift within the industry towards a “quality-driven” transition. Fish muscle quality encompasses multi-dimensional characteristics, including nutritional value (e.g., Essential Amino Acid Index, EAAI; fish lipid quality, FLQ), sensory attributes (texture, flavor), and processing suitability (Carcass Ratio, CR; Cooked Meat Percentage, CMP; Drip Loss Rate, DLR) [8]. These characteristics are subject to complex regulation by multiple factors, including genetics, diet, and environment [8,9,10,11]. Notably, the nutritional composition of fish muscle is not static but dynamically changes with body size [12,13,14], ultimately determining the nutritional value available to consumers. Although nutritional regulation has been demonstrated as one effective approach to improving fish muscle quality [15,16,17,18,19,20,21], existing research exhibits certain limitations: (1) Studies predominantly focus on assessments at a single size, lacking systematic tracking of the dynamic changes in key quality indicators, particularly LC-PUFAs and volatile organic compounds (VOCs), across individuals of varying sizes throughout the entire culture cycle. (2) Existing findings have primarily concentrated on a limited number of fish species, such as rainbow trout (*Oncorhynchus mykiss*) [12,13], tilapia (*Oreochromis niloticus*) [22,23,24], and herring (*Tenualosa ilisha*) [14]. Research on the size-dependent dynamics of quality attributes in economically significant species such as common carp (*Cyprinus carpio* L.) remains severely limited.

The common carp is a globally significant freshwater aquaculture species with immense annual production and constitutes a major economic resource [25]. Optimizing its muscle quality directly affects the ability to meet consumer health demands and enhance industry profitability. However, research on common carp has long focused on genetic improvement, aquaculture techniques, fundamental nutritional requirements, and disease control [26,27,28,29], while systematic investigations into the dynamic changes in key muscle nutrients, sensory attributes, and processing characteristics across individuals of varying sizes remain lacking. To address this gap, a 30-week feeding trial was conducted with common carp. Differences in muscle nutrient composition, sensory attributes, and processing characteristics were systematically tracked among small (SYRC), medium (MYRC), and large-sized (HYRC) individuals. The objectives are (1) to comprehensively elucidate the evolution of core muscle quality indicators—specifically LC-PUFA profiles, textural properties, and VOCs—with increasing body size; (2) to quantitatively compare differences in these core quality attributes across size groups.

## 2. Materials and Methods

### 2.1. Experimental Diets, Fish, and Feeding Procedure

Following conventional feed formulation, a diet containing 5.5% lipids (primarily from soybean oil) and 33.5% crude protein (from soybean meal, rapeseed meal, and cotton meal) was formulated. The diet was prepared and stored following the methodology described in previous studies [30], with its full composition detailed in Table S1. Briefly, raw materials were ground and passed through a 60-mesh screen, mixed according to the principle of small batches and gradual homogenization, extruded into pellets via a twin-screw extruder, dried in ventilated shade, sealed, and stored at −20 °C. A total of 320 common carp (initial weight 11.5 g) with uniform genetic background, size, and health were randomly assigned and equally divided into four replicate net cages (2.0 m × 2.0 m × 1.5 m) for a 30-week trial. Fish were fed three times daily (07:00, 11:30, and 17:30) at a rate of 3% of wet body weight. Water parameters were maintained within the following ranges: dissolved oxygen ≥ 5.0 mg/L, temperature 25.5 ± 3.5 °C, pH 7.4–8.6, and total ammonia nitrogen < 0.05 mg/L.

### 2.2. Experimental Sample Collection and Growth Performance Evaluation

Samples were collected at three body sizes of the farming cycle: small (SYRC, ~80 g, the 10th week), middle (MYRC, ~270 g, the 20th week), and large (HYRC, ~615 g, the 30th week). All fish were starved for 24 h and anesthetized using MS222 (100 mg/L; CAS 886-86-2, Shanghai Aoding Biochemical Technology Co., Ltd., Shanghai, China) until the cessation of opercular movement. Final body weight (FBW), weight gain rate (WGR), feed conversion ratio (FCR), and condition factor (CF) were calculated from body weight and count data. Blood was drawn from the caudal vein of six randomly selected fish per cage using sterile 1 mL syringes. After clotting at 4 °C for 12 h, samples were centrifuged (4 °C, 3500× *g*, 10 min). The serum was aliquoted into 200 μL microcentrifuge tubes and stored at −80 °C. In addition, the dorsal muscles of the fish were dissected and transferred to 15 mL centrifuge tubes. These samples were then flash-frozen in liquid nitrogen and immediately transferred to −80 °C for storage until subsequent analysis of fatty acids (FAs), amino acids, and VOCs. Six fish from each cage were randomly selected for carcass weight measurement (head, fins, and viscera removed), and dorsal muscles were collected and stored at −20 °C for proximate composition analysis (moisture, crude protein, crude lipid, ash). Finally, three fish were randomly selected from each cage for texture profile analysis (TPA) and key physicochemical analysis (pH, CR, CMP, DLR). All muscle analyses focused on a defined section of the dorsal muscle, with strict maintenance of identical anatomical coordinates across body size groups. The standard formulas used were calculated as follows:

WGR (%) = (final body weight − initial body weight)/initial body weight × 100;
FCR = feed intake (dry matter)/fish wet weight gain;
CF = body wet weight (g)/body length (cm)^3^ × 100.

### 2.3. Biochemical Analysis

The following indices were quantified using commercial kits from the same company (Nanjing Jiancheng Bioengineering, Nanjing, China), including triglycerides (TAG, Kit# A110-1-1), total cholesterol (T-CHO, Kit# A111-1-1), high-density lipoprotein cholesterol (HDL-C, Kit# A112-1-1), and low-density lipoprotein cholesterol (LDL-C, Kit# A113-1-1). Briefly, the frozen serum samples were thawed on ice, and the contents of TAG, T-CHO, LDL-C, and HDL-C were detected by micro-methods according to the kit instructions.

### 2.4. Analysis of Proximate Composition

All assays followed AOAC official methods with modifications [31]. Briefly, the content of moisture was determined by drying at 105 °C in a forced-air oven (Memmert UFE600, Mettler-Toledo Technology (China) Co., Ltd., Shanghai, China) until constant weight was reached (±0.001 g/30 min, Mettler Toledo XS205, Mettler-Toledo Technology (China) Co., Ltd., Shanghai, China). The protein content was determined by digesting with concentrated H_2_SO_4_ and catalyst (K_2_SO_4_: CuSO_4_ = 15:1) at 420 °C for 1 h, followed by distillation with 40% NaOH, trapping in H_3_BO_3_ solution (2%, *m*/*v*), and titrating with 0.1 M HCI solution. Crude lipid was quantified by Soxhlet extraction using diethyl ether as solvent. Furthermore, the content of ash was determined by burning the samples in a muffle furnace at 550 °C after carbonization until constant weight was achieved (±0.001 mg). All chemical reagents—H_2_SO_4_, CuSO_4_, NaOH, H_3_BO_3_, HCl, Na_2_CO_3_, and diethyl ether—were of analytical grade and supplied by Tianjin Deen Chemical Reagent Co., Ltd. (Tianjin, China).

### 2.5. Analysis of FA Composition

The samples (0.05–0.1 g) were precisely weighed and homogenized in 8 mL of chloroform–methanol solution (2:1, *v*/*v*) containing 0.1 mL of C17:0 internal standard solution (1 mg/mL). The mixture was vigorously vortexed for 1 min and left at 4 °C for 12 h. After centrifugation (4 °C, 3000 rpm/min, 10 min), 6 mL of supernatant was transferred to Tube II. The residue was re-extracted with 5 mL of chloroform–methanol solution (2:1, *v*/*v*), vortexed, and centrifuged under identical conditions. An additional 3 mL supernatant was collected and combined with Tube II. To the pooled supernatant in Tube II, 2 mL of CaCl_2_ solution (1.6%, *w*/*v*) was added. The mixture was thoroughly agitated and centrifuged (4 °C, 3000 rpm/min, 10 min). The lower phase (3 mL) was transferred to Tube III and evaporated to dryness under a nitrogen stream to obtain lipids. For fatty acid methylation, the lipids were dissolved in 0.3 mL of chloroform and reacted with 1 mL of H_2_SO_4_-methanol solution (2.5%, *v*/*v*) at 70 °C for 1 h in a temperature-controlled water bath. After cooling to room temperature, 0.6 mL of n-hexane and 1.5 mL of ultrapure water were added. The mixture was vortexed and centrifuged (4 °C, 3000 rpm/min, 10 min). The upper organic layer was passed through a 0.22 μm organic filter into a GC vial [32]. Chloroform, methanol, and n-hexane, all of chromatography grade, were supplied by Shanghai Aoding Biochemical Technology Co., Ltd. (Shanghai, China). CaCl_2_ and H_2_SO_4_ were supplied by Tianjin Deen Chemical Reagent Co., Ltd. (Tianjin, China). FAMEs were analyzed by GC-FID (Agilent 7890B, Agilent, Santa Clara, CA, USA) under the following conditions [33]:

Column: DB-WAX (15 m × 0.25 mm × 0.25 μm, Agilent, Santa Clara, CA, USA);
Oven program: 120 °C (hold 2 min) → 4 °C/min → 250 °C (hold 5 min);
Injector: 250 °C, split ratio 10:1;
Detector (FID): 250 °C;
Gas flows (mL/min): Carrier (N_2_): 1.4|Makeup (N_2_): 25|H_2_: 40|Air: 400.

FA species were identified by retention time matching against a 37-component FAME mix (MilliporeSigma, St. Louis, MO, USA) and quantified using the C17:0 internal standard (MilliporeSigma, St. Louis, MO, USA) [34]. The nutritional value assessment formula was as follows:

FLQ = (22:6 n-3 + 20:5 n-3)/Σ FA;
IA = [12:0 + (4 × 14:0) + 16:0]/UFA;
IT = (14:0 + 16:0 + 18:0)/[(0.5 × MUFA) + (0.5 × n-6 PUFA) + (3 × n-3 PUFA) + (n-3/n-6)];
HH = (18:1 + PUFA)/(12:0 + 14:0 + 16:0);
HPI = UFA/[12:0 + (4 × 14:0) + 16:0].

### 2.6. Analysis of AA Composition

Muscle samples (100 mg) were hydrolyzed in sealed tubes with 10 mL of HCl (6 mol/L) under a nitrogen atmosphere at 110 °C for 24 h. After cooling to room temperature, hydrolysates were filtered through quantitative filter paper and diluted to 50 mL with distilled water. Aliquots (2 mL) were transferred to glass tubes, dried under a nitrogen stream at 60 °C, and reconstituted in 2 mL of sodium citrate buffer (Sinopharm Chemical Reagent Co., Ltd., Shanghai, China). Solutions were filtered (0.22 μm nylon membrane) before analysis by an automated amino acid analyzer (Hitachi L-8900, Hitachi High-Tech, Tokyo, Japan) with a 20 μL injection volume. Amino acid nutritional indices [1,35,36] (Amino Acid Score, AAS; Chemical Score, CS; Essential Amino Acid Index, EAAI; Nutrient Index, NI; Predicted Biomass Value, P-BV) were calculated as follows (Table S2):

AAS = AA content of the sample to be tested (mg/g)/AA content of the same AA in the FAO/WHO scoring model (mg/g) × 100;
CS = AA content of the sample to be tested (mg/g)/reference protein AA content of FAO (1970) [37] whole egg amino acid profile (mg/g) × 100;
EAAI = (S1 × S2 × ... × Sn)1/*n*;
NI = EAAI × protein mass fraction;
P-BV= 1.09 × EAAI − 11.7.

Note: *n*: number of EAAs; S1, S2 ... Sn: AAS of each EAA in the sample.

### 2.7. Analysis of Muscle Texture Characterization and Physicochemical Indices

Texture and physicochemical indices were analyzed following Mi et al. [38], with modifications for small muscle samples. A dorsal muscle sample (1.0 cm × 1.0 cm × 0.5 cm; fiber orientation parallel to compression axis) was heated at 95 °C for 5 min, then air-cooled to 25 °C. Texture profile analysis (TPA) was performed using a texture analyzer (Model CT3, Brookfield Engineering Laboratories, Middleboro, MA, USA) with an 8 mm aluminum cylinder probe. Settings: Pre-test speed, 5 mm/s; test speed, 2 mm/s; trigger force, 5 g; 50% deformation; 5 s inter-compression interval. For pH, 1 g of muscle was homogenized with 9 mL of deionized water (10,000 rpm, 1 min, 4 °C), then measured by a pH meter (Mettler-Toledo Technology (China) Co., Ltd., Shanghai, China). The DLP was determined by refrigerating fresh muscle samples (initial mass W_1_) at 4 °C/85% RH for 24 h, followed by surface moisture removal via filter paper and reweighing (W_2_). For CMP analysis, individual fresh muscle samples (initial mass, W_3_) were steamed (100 °C, 5 min), air-cooled to 25 ± 1 °C, and reweighed (W_4_). The calculation formulas were as follows:

DLP (%) = 100 × (W_2_ − W_1_)/W_1_.
CMP (%) = 100 × W_4_/W_3_.

### 2.8. Identification and Analysis of VOCs

VOCs were analyzed by HS-GC-IMS (Shandong Haineng HS-IMS-100, Shandong, China). Finely minced muscle (2.0 ± 0.01 g) was sealed in 20 mL headspace vials and incubated at 60 °C for 15 min. The headspace gas was auto-injected into the GC-IMS. GC conditions: Column, MXT-5 (15 m × 0.53 mm, 1 μm); carrier gas, N_2_ (≥99.999%); gradient flow, 2 mL/min (0–2 min) → 20 mL/min (2–20 min); oven, 60 °C isothermal; syringe, 85 °C; injector: 45 °C. IMS conditions: temperature, 45 °C; drift tube, 9.8 cm; drift gas, N_2_; gas flow rate, 150 mL/min. Retention indices (RIs) for VOCs were calibrated using C_4_-C_9_ n-alkenone standards (Sinopharm Chemical Reagen Co., Ltd., Shanghai, China), with all samples and standards analyzed together under identical GC-IMS conditions to ensure comparability [39].

### 2.9. Calculations and Statistical Analysis

The Shapiro–Wilk and Levene methods were used to test all data for normality and homogeneity of variances. Thereafter, data were analyzed by one-way analysis of variance (ANOVA) using SPSS Statistics (version 22.0; IBM Corp., Armonk, NY, USA), with statistical significance defined at *p* < 0.05. Results are expressed as mean ± standard error of the mean (SEM, *n* = 4). Each sample was analyzed in triplicate (technical replicates) to ensure data reproducibility. To visualize results, graphs were generated using GraphPad Prism (version 9.0; GraphPad Software, San Diego, CA, USA). Additionally, clustering heatmaps of AA profiles were constructed using the Metware Cloud platform (Wuhan MetWare Biotechnology Co., Ltd., Wuhan, China; https://cloud.metware.cn, accessed on 9 March 2025).

## 3. Results

### 3.1. Growth Performance and Serum Biochemical Indices

Growth performance and serum biochemical data are illustrated in Table 1. FBW was significantly increased with increasing fish size, whereas WGR significantly decreased (*p* < 0.05). Both FCR and CF were significantly higher in MYRC than in SYRC and HYRC (*p* < 0.05). In serum biochemistry, LDL-C was significantly elevated in MYRC compared to SYRC and HYRC (*p* < 0.05). In contrast, TAG, T-CHO, and HDL-C concentrations were significantly higher in HYRC and MYRC compared to SYRC (*p* < 0.05). The HDL-C/LDL-C ratio was significantly higher in HYRC than in SYRC and MYRC (*p* < 0.05).

### 3.2. The Proximate Composition, Physicochemical, and Textural Characteristics

**Figure 1:**
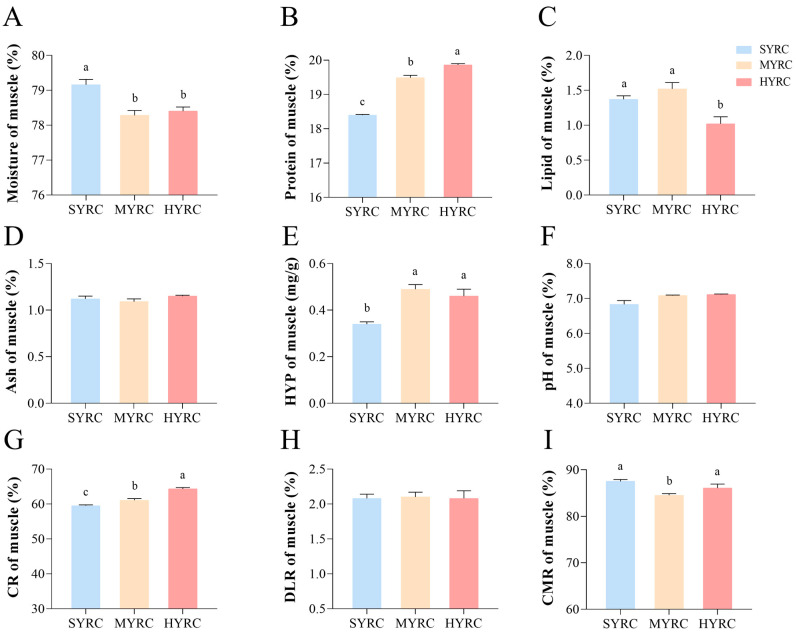
Proximate composition (% wet weight) and physicochemical indexes in muscles of common carp at different sizes ((**A**): moisture; (**B**): protein; (**C**): lipid; (**D**): Ash; (**E**): hydroxyproline; (**F**): pH; (**G**): Carcass Ratio; (**H**): Drip Loss Rate; (**I**): Cooked Meat Percentage). SYRC: small size group; MYRC: middle size group; HYRC: large size group. No identical letters above the columns indicates significant differences between groups (*p* < 0.05); values are mean ± SEM (*n* = 4). CR: Carcass Ratio; CMP: Cooked Meat Percentage; DLR: Drip Loss Rate; HYP: hydroxyproline.

**Figure 2:**
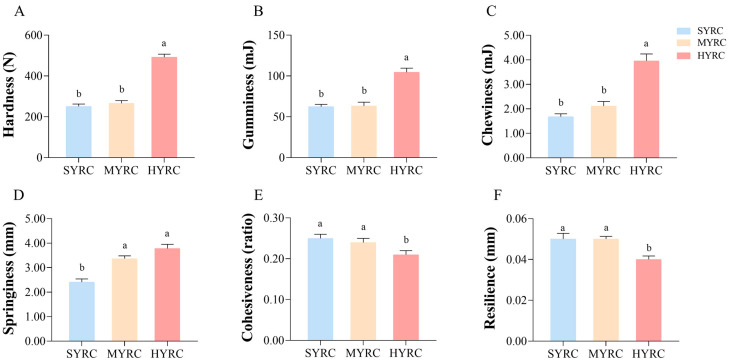
Textural characteristics of muscle in the common carp at different sizes ((**A**): hardness; (**B**): gumminess; (**C**): chewiness; (**D**): springiness; (**E**): cohesiveness; (**F**): resilience). SYRC: small size group; MYRC: middle size group; HYRC: large size group. No identical letters above the columns indicates significant differences between groups (*p* < 0.05); values are mean ± SEM (*n* = 4).

Figure 1 illustrates the proximate composition and physicochemical indices. The results demonstrated a significant reduction in the moisture content (Figure 1A) in MYRC and HYRC compared to SYRC (*p* < 0.05). The content of protein (Figure 1B) and CR (Figure 1G) significantly increased with body size, reaching the highest level in HYRC (*p* < 0.05). The content of lipids (Figure 1C) in SYRC and MYRC was significantly higher than in HYRC (*p* < 0.05). CMP (Figure 1I) was significantly elevated in HYRC and SYRC relative to MYRC (*p* < 0.05). The content of hydroxyproline (HYP, Figure 1E) in HYRC and MYRC was significantly higher than in SYRC (*p* < 0.05). No significant differences were observed in Ash (Figure 1D), DLR (Figure 1H), or pH (Figure 1F) across size groups (*p* > 0.05). As shown in Figure 2, HYRC displayed enhanced texture characteristics than SYRC and MYRC, with higher hardness (Figure 2A), gumminess (Figure 2B), and chewiness (Figure 2C) compared to SYRC and MYRC (*p* < 0.05). Conversely, cohesiveness (Figure 2E) and resilience (Figure 2F) were significantly lower in HYRC than in SYRC and MYRC (*p* < 0.05).

### 3.3. Muscle FA Composition and Nutritional Quality Assessment

In muscle tissue (Table 2), the contents of 14:0, 18:1n-9, 20:1n-9, MUFAs, and C18 PUFAs were significantly higher in MYRC than in SYRC and HYRC (*p* < 0.05). In contrast, key functional FAs, including 20:3n-6, 20: 4n-6 (ARA), 22:6n-3 (DHA), and LC-PUFAs, were significantly enriched in SYRC and HYRC relative to MYRC (*p* < 0.05). The content of 16:0 was significantly lower in HYRC than in MYRC (*p* < 0.05). However, levels of 18:0, SFAs, n-6 PUFAs, and n-3 PUFAs were not significantly different among the different sizes of common carp (*p* > 0.05). Furthermore, in terms of muscle fatty acid nutritive value assessment, FLQ was significantly higher in SYRC and HYRC than in MYRC (*p* < 0.05). HPI and HH were significantly higher in MYRC and HYRC than in SYRC, while IA exhibited the opposite trend (*p* < 0.05). Additionally, n-3/n-6 PUFA levels were found to be significantly higher in SYRC compared to MYRC (*p* < 0.05), while there were no significant differences between SYRC and HYRC (*p* > 0.05). There were no statistically significant differences in PUFA/SFA levels and IT among the different sizes (*p* > 0.05).

### 3.4. Amino Acid Profile and Nutrient Index of Muscle

**Figure 3:**
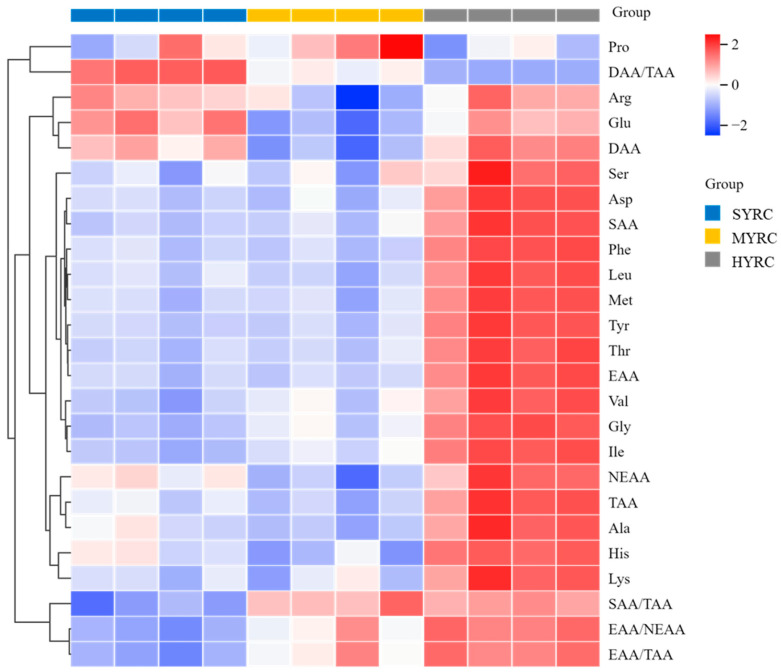
Clustering heat map of muscle AA composition of common carp at different sizes. The degree of enrichment is from low (blue) to high (red), and the trend of similar change is clustered. The color group to the right of the heat map provides a visual guide. EAA: essential amino acid; NEAA: non-essential amino acid; DAA: delicious amino acid; SAA: sweet amino acid; TAA: total amino acid.

Cluster analysis of muscle AA composition is presented in Figure 3. HYRC exhibited significantly elevated levels of essential amino acids (EAAs: Met, Val, Lys, Phe, Leu, and Thr), ΣEAAs, non-essential amino acids (NEAAs: Tyr, Ser, Ala, and Asp), sweet amino acids (SAAs), and total amino acids (TAAs) compared to SYRC and MYRC (*p* < 0.05). The contents of Ile and Gly, as well as EAA/NEAA and EAA/TAA, significantly increased with body size, with the highest values observed in HYRC (*p* < 0.05). The contents of Glu, Arg, and umami amino acids (DAAs) were significantly elevated in HYRC and SYRC relative to MYRC (*p* < 0.05). However, there was a significant decrease in DAA/TAA with the increase in fish size (*p* < 0.05). The ratio of SAA/TAA was significantly higher in HYRC and MYRC than in SYRC (*p* < 0.05).

### 3.5. VOC Profiles of Muscle

**Figure 4:**
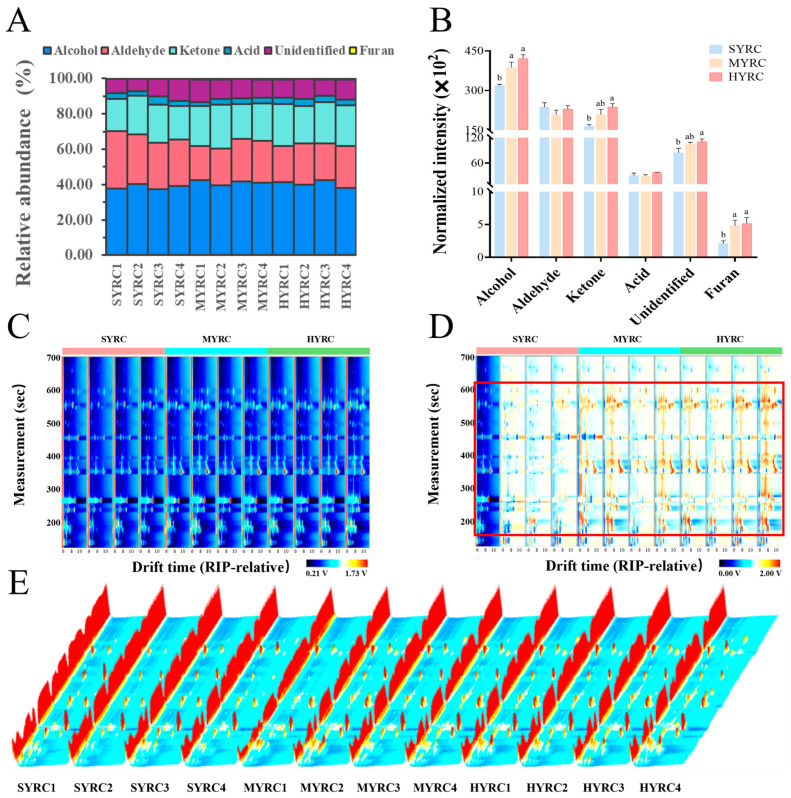
Information on the VOCs in the muscle of common carp at different sizes. Percentage (**A**), concentrations (**B**), two-dimensional spectrum (**C**), difference mapping (**D**), and three-dimensional spectrum (**E**) of VOCs. No identical letters above the columns indicates a statistically significant difference (*p* < 0.05); values are mean ± SEM (*n* = 4). The red square indicate differences in VOCs in the muscles of different groups of Yellow River carp.

In total, 48 VOCs were identified in common carp, including 1 furan, 2 acids, 11 ketones, 16 aldehydes, 16 alcohols, and 2 unidentified compounds (Table 3). Alcohols, ketones, and aldehydes constituted the predominant VOC classes (Figure 4A). As demonstrated in Figure 4B, the levels of alcohols and furans were significantly higher in MYRC than in SYRC (*p* < 0.05), but no difference was observed between HYRC and MYRC (*p* > 0.05). HYRC exhibited significantly higher alcohols, ketones, furans, and unidentified compounds than SYRC (*p* < 0.05). VOC profiles were visualized in two-dimensional (Figure 4C) and three-dimensional (Figure 4E) views. Each point or array of dots in these figures represents a specific VOC. Differential analysis using SYRC1 as the reference revealed that MYRC and HYRC had higher VOC levels (red regions in Figure 4D) than SYRC (*p* < 0.05).

**Figure 5:**
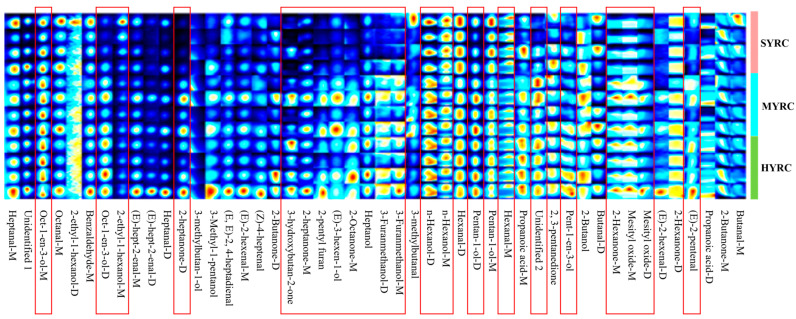
The fingerprint gallery plot of VOCs in the muscle of common carp at different sizes. The red square highlights differential VOCs identified in the muscle tissues of Yellow River carp across experimental groups.

To further elucidate the disparities in the composition of VOCs among different sizes of common carp, the fingerprints of VOCs were constructed based on GC-IMS peak volumes (Figure 5). The levels of 2-heptanone-D (dimer), 2-heptanone-M (monomer), 2-hexanone-M, pentan-1-ol-D, 3-furanmethanol-D, 3-furanmethanol-M, oct-1-en-3-ol-D, oct-1-en-3-ol-M, n-hexanol-D, mesityl oxide-D, heptanol, (E)-3-hexen-1-ol, and 2-pentyl furan were significantly higher in both HYRC and MYRC than in SYRC (*p* < 0.05). A comparison of the levels of 3-hydroxybutan-2-one and 2-octanone-M were significantly higher in HYRC than in SYRC (*p* < 0.05). Similarly, the levels of mesityl oxide-M, pent-1-en-3-ol, and (E)-2-pentenal were found to be significantly higher in HYRC than in MYRC and SYRC (*p* < 0.05). Furthermore, there is a significant increase in the level of ethyl-1-hexanol-M in MYRC compared to HYRC, and SYRC exhibited higher levels of n-Hexanol-M and Hexanal compared to MYRC (*p* < 0.05). Statistically, these differential VOCs included 13 alcohols, five ketones, two aldehydes, one furan, and one unidentified compound (detailed in Table 4).

## 4. Discussion

During aquaculture, significant differences exist in the body composition of fish belonging to different body size categories [40]. In the present study, analysis of muscle quality indices across major size groups of common carp (SYRC, MYRC, HYRC) revealed a significant size-dependent changes in muscle composition during the culture period. Larger fish (HYRC) show progressive protein accretion alongside significant depletion of both lipid and moisture content (*p* < 0.05). This compositional shift aligns with observations from the full-cycle culture of Pacific bluefin tuna (*Thunnus orientalis*) [41] and *Mystus bleekeri* [42]. Such changes likely arise from multifactorial interactions, with dietary intake, metabolic intensity, and locomotor activity serving as primary determinants [43]. Crucially, all experimental fish received the same diet throughout the trial. Meanwhile, increased locomotor activity was observed in HYRC during the later culture stages. This elevated activity likely corresponds to increased metabolic demands in larger, more mature fish. Consequently, the significant depletion of lipid reserves and parallel protein accretion documented in this study may be predominantly attributed to increased energy expenditure associated with greater activity and routine metabolic rates in larger common carp. This interpretation is strongly supported by the work of Wang et al. [44], who demonstrated that hydrodynamic-stimulated exercise in common carp promotes a leaner muscle phenotype, specifically characterized by reduced lipid deposition and elevated protein content.

Beyond elevating nutritional value (EAA, EAA/NEAA, AAS, CS, EAAI, NI, and *p*-BV; Table S3), protein accumulation also modulated key textural attributes such as hardness and chewiness [45]. In this investigation, the HYRC exhibited significantly greater muscle hardness, chewiness, and gumminess compared to SYRC. These textural improvements enhance both organoleptic qualities and processing suitability, boosting the commercial appeal of HYRC. Muscle texture is strongly influenced by collagen content and the stability of its network structure [46,47]. Hydroxyproline, a characteristic amino acid predominantly found in collagen with relatively stable composition, serves as a proxy for collagen quantification. In the present study (Figure 1), Consistent with the hardness increase, hydroxyproline content was significantly higher in HYRC than SYRC. Furthermore, muscle hardness is modulated by muscle fiber histology, intramuscular lipids, and moisture [48]. While early muscle growth relies on fiber hyperplasia, hypertrophy becomes increasingly important later [49]. Although muscle hardness is generally reported to be inversely related to myofiber diameter and directly proportional to density, the potential contribution of myofiber changes to the observed hardness increase in HYRC remains unclear in the absence of specific fiber data in this study. Crucially, higher intramuscular lipid levels are associated with reduced hardness; a 0.5% lipid decrease reportedly increased hardness in tilapia [50]. In the present study, the HYRC muscle exhibited significant compositional shifts compared to SYRC: a decrease of 0.35% in lipid content and a 0.76% decrease in moisture content. Compared to MYRC, HYRC exhibited a 0.5% lipid decrease and a slight 0.12% moisture increase. Therefore, the concurrent decrease in moisture and lipid content, coupled with the increase in collagen content, likely major contributors of the higher hardness observed in large common carp (HYRC) muscle.

LC-PUFAs, representing core nutritional attributes in fish, exhibited a distinct size-dependent accumulation pattern. As the common carp body size increased (from SYRC to MYRC to HYRC), significant alterations in the fatty acid profile were observed. MUFAs, polyunsaturated fatty acids (PUFAs), and specifically C18 PUFAs were significantly lower in the large (HYRC) and small (SYRC) size groups compared to the medium-sized group (MYRC). Conversely, LC-PUFAs were significantly enriched in both the HYRC and SYRC groups. This pattern was consistently observed across muscle, hepatopancreas, and intestinal tissues, demonstrating that LC-PUFA accumulation exhibits a clear body size dependence. This finding moves beyond the traditional research paradigm focused on single-size nutritional fortification, providing new insights into the developmental regulation of LC-PUFA metabolism in common carp, enabling precise nutritional interventions, and informing strategies for functional fish meat development. The accumulation of LC-PUFA relies on dual pathways: exogenous intake and endogenous biosynthesis [2]. Freshwater fish possess desaturase and elongase enzyme systems capable of converting C18 PUFA substrates, such as linoleic acid (LA) and α-linolenic acid (ALA), into LC-PUFAs [40]. Given the absence of LC-PUFAs in the experimental diet (rich in C18 PUFAs), the observed increase in tissue LC-PUFA levels primarily originated from endogenous synthesis. This enhanced biosynthetic strategy likely represents an adaptive response directly linked to the distinct physiological demands of each size class: supporting tissue development or meeting the energy reserve requirements for reproduction [40,51,52,53,54,55]. For instance, the adequate dietary n-3 LC-PUFA requirement for juvenile Japanese flounder (*Paralichthys olivaceus*) has been determined to be 1.4% [40], whereas the requirement for fish in reproductive stages has been established at 2.1% [56]. During the MYRC stage, C18 PUFAs may be preferentially utilized for energy metabolism rather than channeled into LC-PUFA synthesis.

Regarding nutritional value, indices such as flesh lipid quality (FLQ), the Hypocholesterolemic/Hypercholesterolemic ratio (HH), and the Health Promotion Index (HPI) are commonly used to assess the health benefits of food, with higher values indicating greater nutritional value. Conversely, the Index of Atherogenicity (IA) evaluates the potential risk of atherosclerosis, with lower values denoting higher nutritional quality. In this study, FLQ was significantly higher in both HYRC and SYRC compared to MYRC. Similarly, HPI and HH were significantly higher in HYRC and MYRC compared to SYRC. Notably, IA was significantly lower in SYRC. Collectively, these indices indicate that the fatty acid muscle profile of HYRC possesses superior potential for promoting cardiovascular health, making it particularly suitable for dietary recommendations for elderly populations. From an aquaculture perspective, targeted supplementation of n-3 PUFAs (or LC-PUFAs) during the adult stage (HYRC) offers a dual advantage: it can simultaneously enhance production efficiency (e.g., supporting reproductive performance) and increase the value of the resulting fish meat as a functional food.

There is dynamic interplay between fish tissue amino acid/fatty acid profiles and flavor VOCs [57]. In fish muscle, VOCs primarily originate from lipid oxidation and protein degradation [56]. Our data reveal that the protein content increased while the lipid content decreased. This indicates that the accumulation of VOCs is predominantly attributable to lipid (or fatty acid) oxidation. Lipid oxidation proceeds via enzymatic and non-enzymatic pathways [58]. Enzymatic oxidation, chiefly catalyzed by lipoxygenase (LOX), targets unsaturated fatty acids (UFAs) containing cis-1,4-pentadiene structures. This process generates unstable conjugated hydroperoxides, whose subsequent breakdown yields VOCs [59]. Non-enzymatic autoxidation, driven by factors like oxygen, light, temperature, and humidity, also plays a significant role. Autoxidation is a key contributor to VOCs (aldehydes, alcohols, phenols) in lipid-rich environments [60].

In this study, VOC profiles exhibited significant size-dependence. Total VOC levels, including alcohols, ketones, and furans, were significantly elevated in large-sized common carp. It is noteworthy that VOCs increasing with size include both pleasant compounds (pent-1-en-3-ol, the smell of grass/fruit; 3-Furanmethanol, fruit/special natural sweet; 3-hydroxybutan-2-one, creamy), and key substances that cause the earthy smell of aquatic products, such as oct-1-en-3-ol-D (mushroom, earthy smell). The formation of VOCs is also closely related to the type of FAs present in muscle. Earlier research has indicated that VOCs linked with n-3 PUFAs have a favorable flavor profile, while those associated with n-6 PUFAs frequently emit undesirable odors [61].

Current diets rich in n-6 PUFAs (such as soybean oil), may exacerbate this issue. Prolonged high-linoleic-acid (LA, 18:2n-6) diets risk simultaneously reducing nutrition while promoting the accumulation of undesirable off-flavor VOCs (e.g., earthy/musty notes). Extensive evidence highlights the paramount importance of dietary fatty acid balance for optimizing fish quality, encompassing both nutritional value and sensory attributes [61,62,63]. Cheng et al. discovered that tilapia fed a high n-3/n-6 diet exhibited enhanced flavor by increasing the content of volatile aldehydes and alcohols [61]. Similar results have also been reported in golden pompano (*Trachinotus ovatus*) [64] and tench (*Tinca tinca* L.) [65]. Thus, optimizing dietary fatty acid balance, particularly the n-6/n-3 ratio, is imperative. Strategic feed formulation offers dual benefits: enhancing nutritional quality (n-3 LC-PUFA retention) and improving sensory attributes. This approach addresses key market concerns, including n-3 LC-PUFA loss and pronounced “fishy” off-flavors. This is essential for advancing the production of healthier, more palatable aquatic products and ensuring the sustainable and efficient growth of the aquaculture industry.

## 5. Conclusions

**Figure 6:**
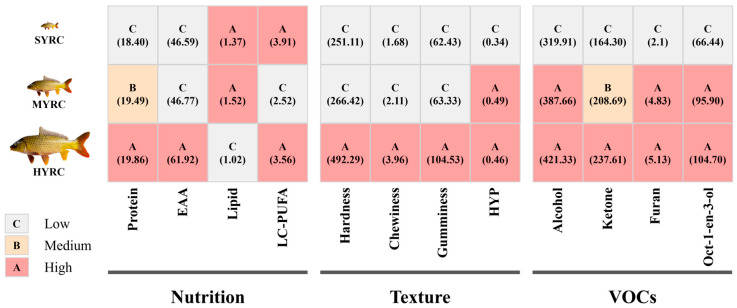
Comprehensive evaluation of common carp muscle quality across different sizes based on nutrition, texture, and VOCs.

In conclusion, this study provides the first comprehensive, multi-scale investigation into the dynamic interplay between common carp body size and critical muscle quality attributes, encompassing nutritional composition, texture, FA metabolism, and VOC profiles. Key findings include (Figure 6): (1) HYRC exhibit increased protein and hydroxyproline content coupled with reduced lipid and moisture levels. These compositional changes were associated with superior textural properties (increased hardness and chewiness) and enhanced nutritional value (primarily due to higher protein content). (2) Both HYRC and SYRC showed higher accumulation of LC-PUFAs compared to MYRC, which exhibited the lowest levels. Moreover, HYRC displayed a lower IA. (3) Critical VOCs, particularly alcohols (e.g., oct-1-en-3-ol and pentan-1-ol), ketones, and furans, accumulate significantly during later culture stages, with key “fishy” flavor compounds like oct-1-en-3-ol from the oxidation of n-6 PUFAs in common carp. Based on the key findings of this study, the following research directions warrant further exploration: (1) developing precision nutrition intervention strategies via functional diets; (2) investigating lipid oxidation (especially enzymatic pathways) and antioxidant systems in body-size-specific VOC production, and identifying off-flavor formation targets in common carp; (3) using multi-omics to elucidate molecular mechanisms underlying size-specific nutrient accumulation and muscle fiber differentiation; (4) developing functional fish products tailored to different populations based on size-specific quality traits. Collectively, unlike previous research limited to a single size point, our multi-size investigation provides empirical evidence for precise nutritional management, targeted muscle quality optimization, and informed consumer guidance, thereby promoting sustainability in common carp aquaculture.

## Figures and Tables

**Table 1:** Growth performance and serum biochemical indexes of different sizes of common carp.

Index	Groups		
	SYRC	MYRC	HYRC
Growth			
IBW (g)	11.62 ± 0.53	66.84 ± 0.93	266.13 ± 1.86
FBW (g)	82.18 ± 2.67 ^c^	263.50 ± 1.10 ^b^	637.07 ± 3.33 ^a^
WGR (%)	707.07 ± 22.97 ^a^	294.22 ± 1.66 ^b^	139.39 ± 1.19 ^c^
FCR	1.36 ± 0.06 ^b^	1.54 ± 0.01 ^a^	1.41 ± 0.01 ^b^
CF (g/cm^3^)	2.39 ± 0.02 ^b^	2.55 ± 0.05 ^a^	2.39 ± 0.02 ^b^
Serum biochemical indexes			
TAG (mmol/L)	2.25 ± 0.18 ^b^	3.58 ± 0.20 ^a^	3.34 ± 0.24 ^a^
T-CHO (mmol/L)	3.43 ± 0.14 ^b^	5.36 ± 0.24 ^a^	4.88 ± 0.22 ^a^
LDL-C (mmol/L)	1.41 ± 0.11 ^b^	2.80 ± 0.15 ^a^	0.76 ± 0.07 ^c^
HDL-C (mmol/L)	1.24 ± 0.08 ^b^	2.34 ± 0.11 ^a^	2.17 ± 0.14 ^a^
HDL-C/LDL-C	0.89 ± 0.03 ^b^	0.85 ± 0.05 ^b^	2.95 ± 0.15 ^a^

Different letters indicate significant differences between different sizes of common carp (*p* < 0.05); values are mean ± SEM (*n* = 4). IBW: initial body weight; FBW: final body weight; WGR: weight gain rate; FCR: feed conversion ratio; CF: condition factor; TAG: triglyceride; T-CHO: total cholesterol; HDL-C: high-density lipoprotein cholesterol; LDL-C: low-density lipoprotein cholesterol.

**Table 2:** FA composition and nutritional quality in the muscle of common carp at different sizes (mg/g).

Index	Groups		
	SYRC	MYRC	HYRC
FA composition			
14:0	0.13 ± 0.01 ^b^	0.26 ± 0.02 ^a^	0.15 ± 0.01 ^b^
16:0	6.40 ± 0.41 ^ab^	7.25 ± 0.43 ^a^	5.86 ± 0.28 ^b^
18:0	2.11 ± 0.12	2.23 ± 0.50	1.95 ± 0.10
20:0	0.03 ± 0.00 ^c^	0.07 ± 0.01 ^a^	0.05 ± 0.00 ^b^
SFA	8.67 ± 0.52	9.80 ± 0.91	8.00 ± 0.39
16:1n-7	0.11 ± 0.02 ^c^	0.89 ± 0.12 ^a^	0.48 ± 0.04 ^b^
18:1n-9	6.73 ± 0.79 ^b^	12.77 ± 0.74 ^a^	7.75 ± 0.49 ^b^
20:1n-9	0.29 ± 0.03 ^b^	0.48 ± 0.03 ^a^	0.38 ± 0.02 ^b^
MUFA	7.14 ± 0.82 ^b^	14.13 ± 0.86 ^a^	8.61 ± 0.54 ^b^
18:2n-6	9.27 ± 0.94 ^b^	12.04 ± 1.13 ^a^	8.90 ± 0.56 ^b^
18:3n-6	0.26 ± 0.02	0.23 ± 0.01	0.22 ± 0.01
20:3n-6	1.04 ± 0.04 ^a^	0.68 ± 0.01 ^c^	0.91 ± 0.02 ^b^
20:4n-6	1.33 ± 0.07 ^a^	1.02 ± 0.09 ^b^	1.45 ± 0.05 ^a^
n-6 PUFA	11.90 ± 1.02	13.97 ± 1.19	11.48 ± 0.58
18:3n-3	0.69 ± 0.08 ^b^	1.20 ± 0.12 ^a^	0.82 ± 0.04 ^b^
20:3n-3	0.01 ± 0.00 ^c^	0.04 ± 0.00 ^a^	0.03 ± 0.00 ^b^
20:5n-3	0.14 ± 0.00 ^a^	0.08 ± 0.01 ^b^	0.07 ± 0.00 ^b^
22:6n-3	1.39 ± 0.10 ^a^	0.71 ± 0.05 ^c^	1.10 ± 0.02 ^b^
n-3 PUFA	2.23 ± 0.09	2.03 ± 0.15	2.03 ± 0.06
LC-PUFA	3.91 ± 0.10 ^a^	2.52 ± 0.16 ^b^	3.56 ± 0.06 ^a^
Nutritional quality			
HPI	3.07 ± 0.10 ^b^	3.63 ± 0.11 ^a^	3.42 ± 0.06 ^a^
FLQ	1.53 ± 0.10 ^a^	0.79 ± 0.06 ^c^	1.17 ± 0.02 ^b^
HH	3.18 ± 0.11 ^b^	3.83 ± 0.13 ^a^	3.54 ± 0.07 ^a^
n-3/n-6	0.19 ± 0.02 ^a^	0.15 ± 0.01 ^b^	0.18 ± 0.01 ^ab^
IA	0.33 ± 0.01 ^a^	0.28 ± 0.01 ^b^	0.29 ± 0.01 ^b^
IT	0.53 ± 0.01	0.48 ± 0.03	0.49 ± 0.01

Different letters indicate significant differences between different sizes of common carp (*p* < 0.05). SYRC: small size group; MYRC: middle size group; HYRC: large size group. Significant differences with different superscript letters were determined at *p* < 0.05; values are mean ± SEM (*n* = 4). SFA: saturated fatty acid; MUFA: monounsaturated fatty acid; PUFA: polyunsaturated fatty acid; LC-PUFA: long-chain polyunsaturated fatty acid; FLQ: fish lipid quality/flesh lipid quality; HPI: health-promoting index; IT: index of thrombogenicity; HH: Hypocholesterolemic/Hypercholesterolemic ratio; IA: Index of Atherogenicity.

**Table 3:** The VOCs in the muscle of common carp at different sizes.

Class	Compound	CAS#	RI	Rt [sec]	Dt [a.u.]
Alcohol (16)	oct-1-en-3-ol-M	C3391864	986.7	555.741	1.1534
	oct-1-en-3-ol-D	C3391864	985	551.777	1.58779
	n-Hexanol-M	C111273	872.8	355.129	1.32201
	n-Hexanol-D	C111273	874.5	357.364	1.65695
	2-ethyl-1-hexanol-M	C104767	1046.5	665.6	1.41635
	2-ethyl-1-hexanol-D	C104767	1045.3	663.335	1.79977
	(E)-3-hexen-1-ol	C928972	855.5	333.891	1.25166
	3-Methyl-1-pentanol	C589355	849.3	326.625	1.30311
	pentan-1-ol-M	C71410	763	237.62	1.24994
	pentan-1-ol-D	C71410	763	237.62	1.52125
	3-methylbutan-1-ol	C123513	735.2	212.453	1.23788
	pent-1-en-3-ol	C616251	682.5	173.319	1.37858
	3-Furanmethanol-M	C4412913	978.7	538.125	1.10407
	3-Furanmethanol-D	C4412913	978.3	537.186	1.34958
	Heptanol	C53535334	976.1	532.495	1.39346
	2-Butanol	C78922	587.6	136.563	1.16311
Aldehyde (16)	Octanal-M	C124130	1011.7	603.309	1.42214
	(E)-hept-2-enal-M	C18829555	957.1	493.577	1.25369
	(E)-hept-2-enal-D	C18829555	958.6	496.525	1.6573
	(Z)-4-heptenal	C6728310	900.8	394.019	1.14015
	(E)-2-hexenal-M	C6728263	851.2	328.861	1.17816
	(E)-2-hexenal-D	C6728263	848.8	326.066	1.50786
	(E, E)-2,4-heptadienal	C4313035	1021.3	619.857	1.19829
	(E)-2-pentenal	C1576870	748.8	224.46	1.10294
	Heptanal-M	C111717	902.4	396.486	1.35351
	Heptanal-D	C111717	904.2	399.281	1.68845
	Hexanal-D	C66251	792.4	266.579	1.55595
	Hexanal-M	C66251	793.7	267.834	1.28602
	Benzaldehyde-M	C100527	963	505.37	1.14816
	butanal-M	C123728	588.8	136.968	1.10489
	butanal-D	C123728	589.9	137.372	1.28822
	3-methylbutanal	C590863	639	155.383	1.18664
Ketone (11)	2-Hexanone-M	C591786	797.3	271.309	1.21854
	2-Hexanone-D	C591786	793	267.161	1.49324
	3-hydroxybutan-2-one	C513860	717.7	197.979	1.32651
	2,3-pentanedione	C600146	690.5	177.349	1.22624
	2-Butanone-M	C78933	588.6	136.904	1.0781
	2-Butanone-D	C78933	589.6	137.248	1.24155
	2-heptanone-M	C110430	891.5	379.72	1.26111
	2-heptanone-D	C110430	892	380.279	1.6181
	2-Octanone-M	C111137	996.2	577.26	1.33063
	Mesityl oxide-M	C141797	800.4	274.272	1.12174
	Mesityl oxide-D	C141797	795.5	269.532	1.42392
Furan (1)	2-pentyl furan	C3777693	996.2	577.26	1.23912
Acid (2)	Propanoic acid-M	C79094	712.7	193.96	1.10182
	Propanoic acid-D	C79094	713.5	194.576	1.27144
Unidentified (2)	unidentified 1	-	987	556.307	1.43952
	unidentified 2	-	715.4	196.135	1.16306

CAS#: chemical abstracts service number; RI: retention index; RT [sec]: retention time; DT [a.u.]: detector time.

**Table 4:** Differential VOCs in muscles of common carp at different stages.

Compound	Class	Groups		
		SYRC	MYRC	HYRC
2-heptanone-D	Ketone	391.70 ± 70.55 ^b^	1639.99 ± 271.92 ^a^	1500.31 ± 291.76 ^a^
2-heptanone-M	Ketone	654.17 ± 82.12 ^b^	1035.02 ± 63.58 ^a^	981.47 ± 73.53 ^a^
2-Hexanone-M	Ketone	1955.30 ± 72.47 ^b^	2325.51 ± 76.84 ^a^	2306.75 ± 38.12 ^a^
3-hydroxybutan-2-one	Ketone	1536.04 ± 664.20 ^b^	3406.34 ± 1086.37 ^ab^	5189.92 ± 652.16 ^a^
2-Octanone-M	Ketone	190.04 ± 15.09 ^b^	321.02 ± 48.82 ^ab^	408.47 ± 53.80 ^a^
pentan-1-ol-D	Alcohol	2470.53 ± 420.17 ^b^	4711.10 ± 556.58 ^a^	4846.99 ± 397.81 ^a^
3-Furanmethanol-D	Alcohol	140.10 ± 29.77 ^b^	274.11 ± 29.23 ^a^	304.27 ± 21.12 ^a^
3-Furanmethanol-M	Alcohol	577.11 ± 94.41 ^b^	932.40 ± 67.57 ^a^	1135.35 ± 65.62 ^a^
oct-1-en-3-ol-D	Alcohol	1257.79 ± 272.53 ^b^	2628.42 ± 290.25 ^a^	3134.26 ± 217.34 ^a^
oct-1-en-3-ol-M	Alcohol	5386.57 ± 541.89 ^b^	6961.14 ± 151.95 ^a^	7336.22 ± 164.75 ^a^
n-Hexanol-D	Alcohol	4632.20 ± 269.15 ^b^	7455.97 ± 241.84 ^a^	7439.56 ± 285.79 ^a^
Mesityl oxide-D	Alcohol	2275.51 ± 138.51 ^b^	2934.47 ± 223.47 ^a^	2932.77 ± 159.07 ^a^
Heptanol	Alcohol	514.50 ± 39.56 ^b^	803.34 ± 59.54 ^a^	867.75 ± 81.78 ^a^
(E)-3-hexen-1-ol	Alcohol	119.68 ± 16.78 ^b^	241.13 ± 31.24 ^a^	174.71 ± 16.38 ^a^
Mesityl oxide-M	Alcohol	1539.25 ± 71.83 ^b^	1611.87 ± 135.17 ^b^	1915.81 ± 23.83 ^a^
pent-1-en-3-ol	Alcohol	2049.83 ± 285.57 ^b^	2614.82 ± 430.85 ^b^	4005.95 ± 456.65 ^a^
2-ethyl-1-hexanol-M	Alcohol	661.13 ± 98.68 ^a^	480.14 ± 10.63 ^ab^	461.1.59 ^b^
n-Hexanol-M	Alcohol	5619.22 ± 201.04 ^a^	4795.00 ± 275.89 ^b^	5253.34 ± 148.38 ^ab^
(E)-2-pentenal	Aldehyde	358.07 ± 62.82 ^b^	399.32 ± 41.56 ^b^	573.42 ± 54.64 ^a^
Hexanal-M	Aldehyde	2553.37 ± 36.42 ^a^	1915.24 ± 177.62 ^b^	2160.42 ± 129.08 ^ab^
2-pentyl furan	Furan	183.25 ± 38.88 ^b^	483.40 ± 82.19 ^a^	513.18 ± 91.91 ^a^
unidentified 2	-	2433.63 ± 459.98 ^b^	4227.97 ± 326.38 ^a^	4514.08 ± 94.71 ^a^

Different letters indicate significant differences between different sizes of common carp (*p* < 0.05); values are mean ± SEM (*n* = 4).

## Data Availability

The original contributions presented in this study are included in the article/Supplementary Materials. Further inquiries can be directed to the corresponding authors.

## Supplementary Materials

The following supporting information can be downloaded at: https://www.mdpi.com/article/10.3390/foods14162794/s1, Table S1: Raw material composition, proximate composition, and major fatty acid composition of basal diet; Table S2: The EAA requirement is included in the FAO/WHO and the high-quality egg protein pattern spectrum (mg/g); Table S3: Evaluation of AAS, CS, EAAI, NI, and P-BV in muscles of Yellow River carp at different sizes.
