# Supplementary material for: Optimizing Muscle Quality in Common Carp (Cyprinus carpio L.): Impacts of Body Size on Nutrient Composition, Texture, and Volatile Profile
Source: Foods. 2025 Aug 11;14(16):2794. doi: 10.3390/foods14162794 (PMC12386061; doi:10.3390/foods14162794)
Supplement: Supplementary file 1 [file foods-14-02794-s001.zip › foods-3762582-supplementary.pdf]

Table S1 Raw material composition, proximate composition, and major fatty acid composition of basal diet

Index	content
**Raw material composition (%)**	
Soybean meal	30.00
Rapeseed meal	13.00
Corn gluten meal	7.00
Cottonseed meal	11.00
Wheat meal	24.25
Soybean oil	4.00
Microcrystalline cellulose	5.77
Calcium dihydrogen phosphate	2.00
Choline chloride	0.20
Mixed vitamins[a]	0.07
Mixed mineral[b]	0.10
Lysine	0.90
Methionine	0.71
Bentonite	1.00
**proximate composition (% dry matter)**	
Crude protein	33.58
Crude lipid	5.54
**Main fatty acid composition (% total fatty acids)**	
16:0	13.39
18:1	28.73
18:2n-6	48.14
18:3n-3	5.37

The raw materials were provided by Luohe City, Henan Province, Zhengda Co., Ltd.

[a,b] Supplied by Zhuhai Weinuo Breeding Co., Ltd., Zhuhai, China. Vitamin premix (g kg-1 premix): nicotinamide, 10.00~42.00; inositol, tocopherol, 9.00~30.00; 7.20~24.00; ascorbicacid, 6.00~20.00; pantothenic acid, 5.50~18.00; menadione, 2.70~9.00; folic acid, 0.90~3.00; riboflavin, 0.86~2.88; retinyl acetate, 0.66~1.98; thiamine, 0.55~1.80; cholecalciferol, 0.33~0.66; biotin, 0.02~0.06; cyanocobalamin, 0.01~0.02. Mineral premix (g kg−1 premix): manganese, 10.00~40.00; zinc, 8.00~32.00; iron, 5.00~20.00; copper, 3.00~12.00; iodine, 0.20~0.80; selenium, 0.10~0.40.

Table S2 The EAA requirement is included in the FAO/WHO and the high-quality egg protein pattern spectrum (mg/g)

Compound	Infant (0 m-6 m)	Infant (6 m-3 y)	Children (>3 y), Adolescents, adults	Egg
Met+Cys	33	27	23	57
Val	55	43	40	66
Lys	69	57	48	70
Ile	55	32	30	54
Phe+Tyr	94	52	41	93
Leu	96	66	61	86
Thr	44	31	25	47
His	21	20	16	22

Table S3 Evaluation of AAS, CS, EAAI, NI, and P-BV in muscles of Yellow River carp at different sizes

Model	Class	Index	Groups		
			SYRC	MYRC	HYRC
FAO/WHO	Infant (0 m-6 m)	Met+Cys	90.05±1.88b	89.76±2.55b	117.81±2.18a
		Val	104.59±2.10b	111.66±2.52b	135.22±2.90a
		Lys	146.89±2.75b	146.52±5.12b	182.01±4.25a
		Ile	77.42±1.78c	87.80±2.37b	125.90±2.39a
		Phe+Tyr	79.52±1.64b	78.53±1.84b	113.78±2.32a
		Leu	98.83±1.61b	95.74±1.99b	125.43±2.52a
		Thr	100.97±2.66b	103.06±2.85b	153.95±4.06a
		His	231.19±5.38b	210.45±7.67c	276.56±1.90a
		EAAI	108.80±2.11b	109.63±1.54b	147.31±2.85a
		NI	20.02±0.39b	21.37±0.30b	29.26±0.57a
		P-BV	106.89±2.30b	107.80±1.68b	148.87±3.10a
	Infant (6 m-3 y)	Met+Cys	110.06±2.30b	109.70±3.12b	143.99±2.66a
		Val	133.77±2.69b	142.83±3.22b	172.95±3.71a
		Lys	177.82±3.33b	177.36±6.19b	220.32±5.14a
		Ile	133.06±3.05c	150.90±4.06b	216.40±4.11a
		Phe+Tyr	143.75±2.97b	141.96±3.33b	205.68±4.20a
		Leu	143.76±2.34b	139.25±2.89b	182.45±3.66a
		Thr	143.31±3.78b	146.27±4.04b	218.51±5.76a
		His	242.75±5.65b	220.98±8.05c	290.39±1.99a
		EAAI	149.55±2.90b	150.70±2.11b	202.49±3.91a
		NI	27.52±0.53b	29.37±0.41b	40.21±0.78a
		P-BV	151.31±3.16b	152.56±2.30b	209.01±4.27a
	Children (＞3 y), Adolescents, adults	Met+Cys	129.20±2.69b	128.78±3.66b	169.03±3.12a
		Val	143.81±2.89b	153.54±3.46b	185.93±3.99a
		Lys	211.16±3.95b	210.62±7.36b	261.64±6.11a
		Ile	141.93±3.26c	160.96±4.34b	230.83±4.39a
		Phe+Tyr	182.32±3.77b	180.04±4.23b	260.87±5.32a
		Leu	155.54±2.53b	150.67±3.13b	197.40±3.96a
		Thr	177.71±4.69b	181.38±5.01b	270.95±7.15a
		His	303.44±7.06b	276.22±10.07c	362.98±2.49a
		EAAI	174.27±3.38b	175.61±2.46b	235.96±4.56a
		NI	32.07±0.62b	34.23±0.48b	46.86±0.91a
		P-BV	178.25±3.68b	179.72±2.68b	245.49±4.97a
EGG		Met+Cys	52.13±1.09b	51.96±1.48b	68.21±1.26a
		Val	87.16±1.75b	93.05±2.10b	112.68±2.42a
		Lys	144.80±2.71b	144.43±5.04b	179.41±4.19a
		Ile	78.85±1.81c	89.42±2.41b	128.24±2.44a
		Phe+Tyr	80.38±1.66b	79.37±1.86b	115.01±2.35a
		Leu	110.33±1.80b	106.87±2.22b	140.02±2.81a
		Thr	94.53±2.49b	96.48±2.67b	144.12±3.80a

His	220.68±5.13b	200.89±7.32c	263.99±1.81a
EAAI	99.47±1.93b	100.24±1.41b	134.69±2.60a
NI	18.30±0.35b	19.54±0.27b	26.75±0.52a
P-BV	96.73±2.10b	97.56±1.53b	135.11±2.84a

Different letters indicate significant differences between different sizes of Yellow River carp ($P < 0.05$), values are mean ± SEM (n = 4). EAAI: essential amino acid index; NI: nutrient index; P-BV: predicted biomass value.
